# Comparative Genome-Wide Analysis of MicroRNAs and Their Target Genes in Roots of Contrasting *Indica* Rice Cultivars under Reproductive-Stage Drought

**DOI:** 10.3390/genes14071390

**Published:** 2023-07-01

**Authors:** Simardeep Kaur, Karishma Seem, Suresh Kumar, Rakesh Kaundal, Trilochan Mohapatra

**Affiliations:** 1 Division of Biochemistry, ICAR-Indian Agricultural Research Institute, New Delhi 110012, India; 2 Department of Plants, Soils, and Climate, College of Agriculture and Applied Sciences, Utah State University, Logan, UT 84322, USA; 3 Bioinformatics Facility, Center for Integrated BioSystems, College of Agriculture and Applied Sciences, Utah State University, Logan, UT 84322, USA; 4 Indian Council of Agricultural Research, New Delhi 110012, India

**Keywords:** *Oryza sativa* L., abiotic stress, terminal drought, miRNome profile, novel miRNAs, transcription factor

## Abstract

Recurrent occurrence of drought stress in varying intensity has become a common phenomenon in the present era of global climate change, which not only causes severe yield losses but also challenges the cultivation of rice. This raises serious concerns for sustainable food production and global food security. The root of a plant is primarily responsible to perceive drought stress and acquire sufficient water for the survival/optimal growth of the plant under extreme climatic conditions. Earlier studies reported the involvement/important roles of microRNAs (miRNAs) in plants’ responses to environmental/abiotic stresses. A number (738) of miRNAs is known to be expressed in different tissues under varying environmental conditions in rice, but our understanding of the role, mode of action, and target genes of the miRNAs are still elusive. Using contrasting rice [IR-64 (reproductive-stage drought sensitive) and N-22 (drought-tolerant)] cultivars, imposed with terminal (reproductive-stage) drought stress, we demonstrate differential expression of 270 known and 91 novel miRNAs in roots of the contrasting rice cultivars in response to the stress. Among the known miRNAs, osamiR812, osamiR166, osamiR156, osamiR167, and osamiR396 were the most differentially expressed miRNAs between the rice cultivars. In the root of N-22, 18 known and 12 novel miRNAs were observed to be exclusively expressed, while only two known (zero novels) miRNAs were exclusively expressed in the roots of IR-64. The majority of the target gene(s) of the miRNAs were drought-responsive transcription factors playing important roles in flower, grain development, auxin signaling, root development, and phytohormone-crosstalk. The novel miRNAs identified in this study may serve as good candidates for the genetic improvement of rice for terminal drought stress towards developing climate-smart rice for sustainable food production.

## 1. Introduction

Drought stress is one of the most serious abiotic stresses limiting the growth and productivity of rice; thus, raising serious concerns about global food security [1,2]. Rice plays a critical role as a primary food source for approximately half of the world’s population, supplying around one-fifth of the total caloric intake worldwide [3,4]. Apart from providing nutritious food to the rapidly growing global population, rice is a model crop plant having a fully-sequenced genome, and extensive collections of diverse germplasm, and is used for basic studies on crops [3,5,6,7,8]. However, rice (being semi-aquatic) requires plenty of water for irrigation to attain ample yield and utmost productivity. Moreover, rice is more sensitive to the drought stress occurring at the reproductive stage of growth (terminal drought), as it severely affects panicle initiation, flowering, pollen viability, and grain yield [8,9,10]. As a result, the recurring occurrence of drought poses significant challenges to rice cultivation in the current era of global climate change, leading to substantial yield losses [8,11,12,13,14].

Efforts are being made towards the identification and characterization of diverse mechanisms (including morphological, physiological, biochemical, genetic, and epigenetic) involved in providing drought stress tolerance in rice [14,15]. Drought stress induces alterations in root system architecture (RSA) by modulating cellular hydraulics and/or water permeability [16,17]. Plants adopt various morpho-physiological changes as part of their strategies to mitigate the adverse impacts of drought stress. Some of these adaptations include the development of deeper root systems to enhance the acquisition of soil moisture and reduce transpiration rates from leaves [18,19]. Hence, functional adaptations in roots are some of the important parameters for the survival of plants and grain yield under drought stress [20,21].

Several drought-responsive biochemical, molecular, and epigenetic regulatory mechanisms active against drought stress have been reported [8,11,15,22]. An important recent breakthrough in plant research is the recognition of microRNAs (miRNAs) as key regulators of stress-responsive genes [23,24]. MiRNAs, which are small (19–24 nt) endogenous, single-stranded, non-coding RNAs, play a crucial role in down-regulating gene expression at the post-transcriptional level. [24,25,26,27,28]. Interestingly, miRNA regulates gene expression either by binding to the target mRNA through partial/imperfect complementarity [29] or by cleaving the mRNA [30] resulting in translational repression of the gene. The miRNAs usually target transcription factors (TFs) and crucial drought-responsive genes, which in turn regulate multiple genomic loci involved in drought-adaptive responses of the plant [29,31,32,33,34,35]. As one of the key regulators of gene expression under environmental stresses, miRNA is being reported to be involved in developmental processes including grain yield, productivity, and stress responses [36,37,38,39,40,41,42].

The major classes of TFs that respond during drought stress include AREB/ABF, MYB, Nuclear Factor Y-B (NF-YB), NAC [No apical meristem (NAM) Arabidopsis transcription activation factor (ATAF) Cup-shaped cotyledon CUC)], Ethylene Responsive Factors (ERF), Dehydration Responsive Element Binding (DREB) 2A, and WRKY, which regulate expression of numerous stress-associated genes [24,35,43]. Studies suggest that the expression of the members of MYB, NAC, NF-YB, and NAC TF families are regulated by miRNAs [35,43,44,45,46,47]. Studies also suggest that differential expression of miRNAs under drought stress plays important roles in regulating various developmental and biological processes including growth, development, photosynthesis, respiration, osmotic stress, antioxidant defense, plant growth regulator signaling, and senescence in crop plants [23,24,25,34,43,48,49,50,51,52,53,54].

Grain development requires a series of molecular events dependent on water availability; however, the reproductive stage drought stress significantly reduces water content in the plant affecting seed setting and grain yield. Analysis of the functional significance of miRNA under drought stress has been carried out by several researchers [4,17,35,55,56,57]; however, elucidation of the regulatory networks integrating miRNA-associated machinery involved in drought stress tolerance would be necessary to design miRNA-based strategy for crop improvement. Comprehensive/comparative analysis of the expression of miRNAs and their targets in roots of contrasting rice cultivars under drought stress is the tip of the iceberg. The miRBase dataset contains a total of 738 miRNAs discovered so far expressing in rice under diverse conditions in different tissues, which are considered to be just 2–3% of the miRNAs encoded by the rice genome, indicating that a large number of miRNAs are yet to be identified.

Therefore, the present study was aimed to identify, characterize, and validate the functional significance of miRNAs (known as well as novel) and their target gene(s) in response to the terminal drought stress in the roots of contrasting rice cultivars. We report, the differential expression of 270 known and 91 novel miRNAs in the roots of contrasting rice cultivars in response to terminal drought stress. While 18 known and 12 novel miRNAs were exclusively expressed in roots of N-22, only two known (but none of the novel) miRNAs were exclusively expressed in roots of IR-64 under stress. The majority of the miRNAs targeted drought-responsive TF genes involved in root development, flowering, grain development, auxin signaling, and phytohormone-crosstalk. This information would enhance our understanding of miRNA-mediated gene regulatory networks under drought stress towards the development of climate-resilient rice for sustainable food production and zero-emission agriculture.

## 2. Results

**Figure 1:**
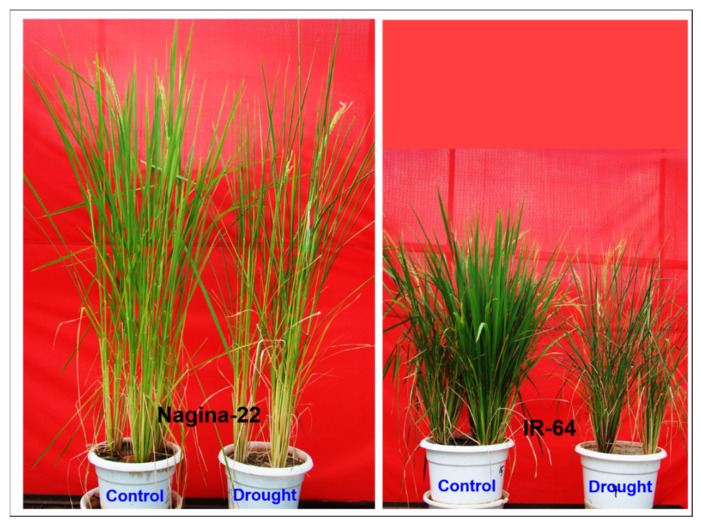
Shoot morphology of contrasting rice (Nagina-22 and IR-64) cultivars (from which root tissues were collected) imposed with reproductive stage (terminal) drought stress by withholding irrigation until the soil moisture content reduced to ~6% and relative water content of leaf dropped down to ~58%.

Imposition of terminal drought stress by withholding irrigation to reduce soil moisture content (SMC) to ~6% (~24% SMC under control) resulted in a significant drop (down to 58 ± 1%) in relative water content (RWC) of the leaf of IR-64, compared to a significantly higher (61 ± 1%) RWC in leaves of N-22. Although a clear-cut morphological change/effect of the drought stress could not be observed on roots, wilting and rolling-off of the leaves could be visualized (Figure 1). This confirmed that the root samples collected from the stress-treated plants for comparative study were from the plants imposed with severe drought stress. 

### 2.1. Processing and Analysis of Small RNA-Seq Data 

To decipher the drought-responsive miRNAs expressed in roots of contrasting rice cultivars under terminal drought stress (soil moisture content ~6% and relative water content of leaf ~58%), small-RNA libraries for roots from the contrasting rice cultivars, N-22 (drought tolerant) and IR-64 (drought sensitive) were prepared in single replication. Sequencing of the sRNA libraries at Illumina HiSeq2500 platform using SE50 bp chemistry generated > 2.6 million reads for each sample (Supplementary Table S1). After filtering the reads to remove adaptor contamination, low-quality reads, reads with <17 or >35 nt sRNA sequence, and >10% unknown bases, only ~0.64 million reads containing sRNA remained for each sample which was used for further bioinformatics analyses (an overview of the methodology used for identification, characterization, and functional validation of the drought-responsive known and novel miRNAs in roots of rice has been provided in the Section 4).

### 2.2. Identification of miRNAs Expressed in the Rice Cultivars 

**Figure 2:**
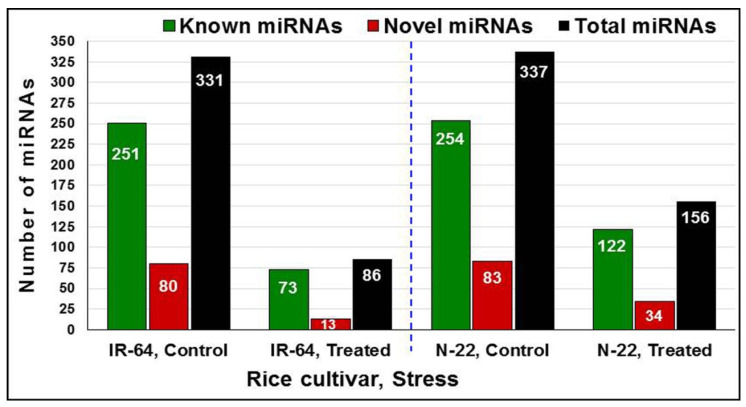
Differential expression of the known, novel, and total miRNAs in contrasting rice cultivars under control and treated [reproductive-stage (terminal) drought stress] conditions.

The objective of the study was to identify the known as well as the novel miRNAs expressed in roots of the contrasting rice cultivars under control and terminal drought stress. The criteria for identification were a minimum expression level of ≥1 transcript per million (TPM) in at least one library. By comparing the identified miRNA sequences with the mature miRNA sequences in rice miRBase 22.1, a total of 296 known miRNAs belonging to 116 different families were found to be expressed in the rice cultivars under control and stressed conditions (Supplementary Table S2). Interestingly, the roots of the N-22 cultivar under control conditions exhibited the highest number of expressed miRNAs, including both known and novel ones. However, under stress, the number of expressed miRNAs decreased to approximately 50% in N-22 and dropped even further to around one-fourth in the case of IR-64, another rice cultivar (Figure 2). These findings highlight the dynamic nature of miRNA expression in response to both environmental conditions and genotypic variations, providing insights into the regulatory mechanisms involved in drought stress responses in rice roots. 

**Figure 3:**
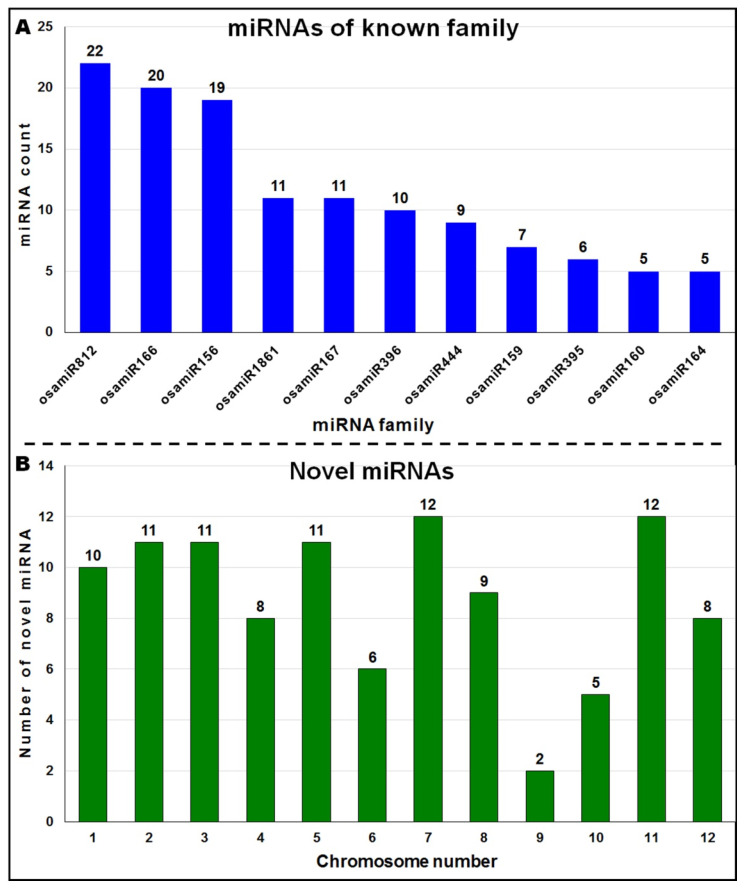
The miRNAs differentially expressed in roots of contrasting rice (N-22 and IR-64) cultivars under control and terminal drought stress. (**A**) The number of miRNA for different known families expressed in roots of the rice cultivars, (**B**) chromosome-wise distribution of novel miRNAs expressed in the root of the rice cultivars under control and terminal drought stress.

The most prevalent miRNA families included miR812, miR166, miR156, miR1861, miR167, and miR396 from which >10 miRNAs were expressed in the roots of the rice cultivars under drought stress (Figure 3A). The sequences which did not show any homology with any known miRNAs in the rice or other plant miRBase were identified as putative novel miRNAs. Such sequences were subjected to miRDeep2 scrutiny for miRNA feature identification including the presence of star (miRNA*) sequence, length of miRNA (19–24 nt), and length of precursor miRNA (>60 nt). The most important criteria to distinguish miRNA from other non-coding RNAs and coding RNAs, such as minimal folding energy (MFE, −23 to −70 kcal/mol) and minimal folding energy index (MFEI, >0.73), were used to screen out the putative novel miRNAs (Supplementary Table S3). Thus, a total of 105 novel miRNAs were identified as predicted miRNAs in two contrasting rice cultivars grown under control and reproductive stage (terminal) drought stress, which were tentatively named Novel-osa-1 to 105. Chromosomal distribution of the novel miRNAs indicated that they were located on all 12 rice chromosomes with the maximum number on chromosomes 7 and 11, and a minimum on 9 (Figure 3B).

**Figure 4:**
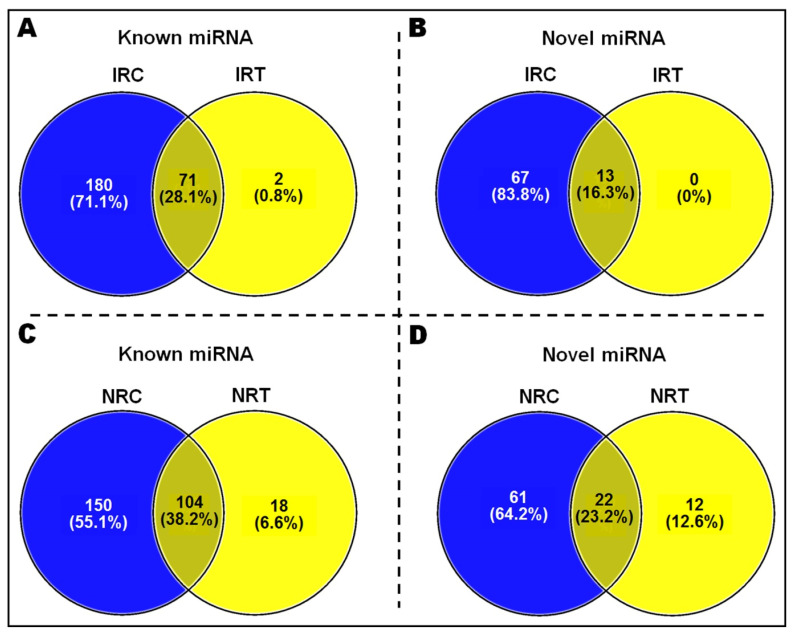
Venn diagrams presenting the number of exclusively and commonly expressed known and novel miRNAs in the contrasting rice cultivars under control and reproductive-stage (terminal) drought stress. (**A**) Known miRNAs commonly and exclusively expressed under control and treatment in IR-64, (**B**) novel miRNAs commonly and exclusively expressed under control and treatment in IR-64, (**C**) known miRNAs commonly and exclusively expressed under control and treatment in N-22, and (**D**) novel miRNAs commonly and exclusively expressed under control and treatment in N-22.

Under the reproductive stage drought stress, 18 known miRNAs were induced exclusively in N-22, while induced expression of only 2 miRNAs was observed exclusively in IR-64. Similarly, 12 novel miRNAs were exclusively expressed in roots of N-22, (while zero novel miRNA were expressed in IR-64), induced by the stress (Figure 4).

### 2.3. Differential Expression of miRNA in Root

**Figure 5:**
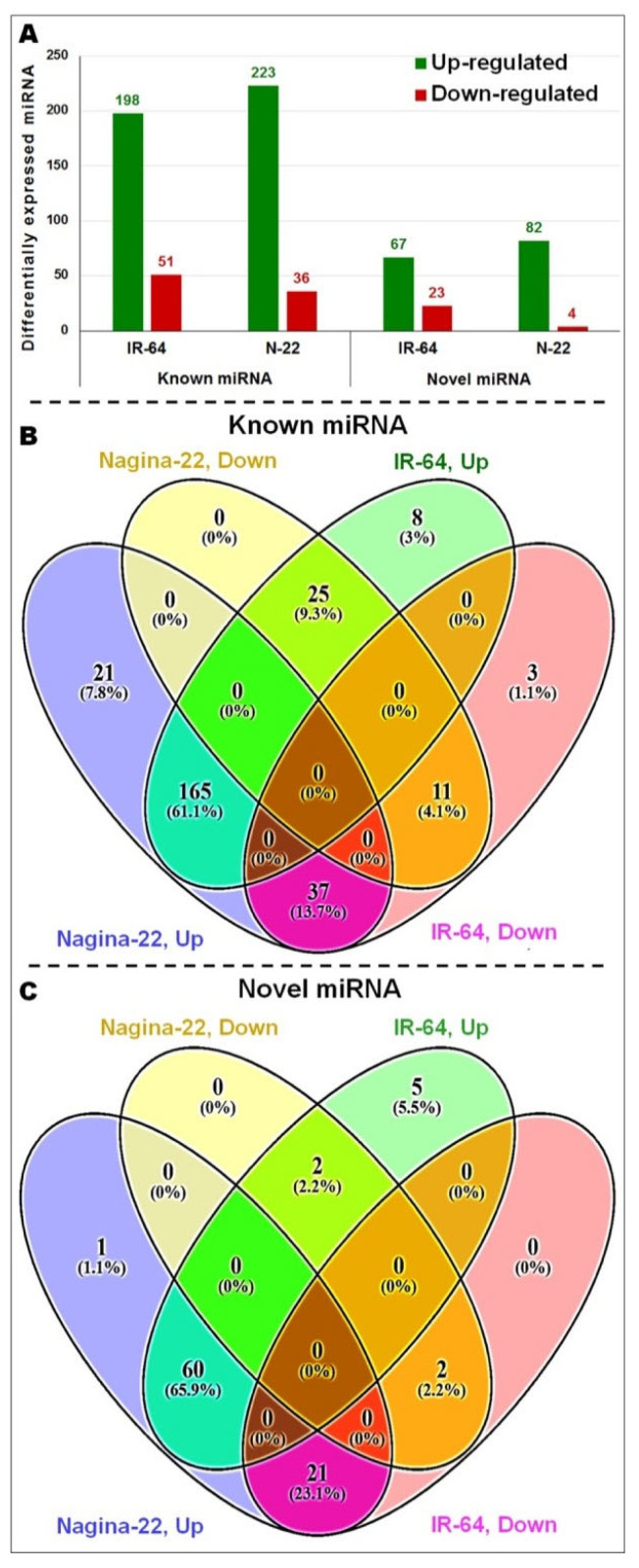
Distribution of differentially expressed known and novel miRNAs in roots of the contrasting rice (N-22 and IR-64) cultivars under reproductive stage (terminal) drought stress. (**A**) Up- and down-regulated known as well as novel miRNAs, (**B**) four way Venn diagram showing distribution of known miRNAs, (**C**) four way Venn diagram showing distribution of novel miRNAs.

To have an in-depth understanding of the differential expression of miRNAs in roots of the contrasting rice cultivars under terminal drought stress, a comparative analysis was performed using normalized data for the predicted novel as well as known miRNAs (≥2-fold change, *p*-value ≤ 0.05) (Supplementary Table S2). Based on the comparative analysis, we could identify a total of 296 known and 105 novel miRNAs differentially expressed in the roots of the contrasting rice cultivars (Supplementary Tables S2 and S3). Interestingly, more up-regulated known (223, Supplementary Table S4) as well as novel (82) miRNAs were expressed in roots of N-22 (drought-tolerant cultivar) compared to that in IR-64 (drought-sensitive cultivar). However, the down-regulated known (51, Supplementary Table S5) as well as novel (23) miRNAs were more in IR-64 (Figure 5A). More interestingly, 21 known miRNAs were exclusively up-regulated in roots of N-22 while 37 of the miRNAs up-regulated in roots of N-22 were down-regulated in IR-64 (Figure 5B). Similarly, 1 novel miRNA was exclusively up-regulated in the roots of N-22, while the 21 novel miRNAs up-regulated in the roots of N-22 were down-regulated in the roots of IR-64 (Figure 5C). Furthermore, 198 known miRNAs were up-regulated in the roots of IR-64 under the stress (Supplementary Table S6), whereas only 36 known miRNAs were down-regulated in the roots of N-22 under the stress (Supplementary Table S7).

### 2.4. Target Prediction for Known and Novel miRNAs in Rice

To identify the target gene of known and novel miRNAs, a target gene prediction program for plant miRNA psRNATarget was used at default parameters, which indicated that 88 drought-responsive novel miRNAs (out of 105 predicted novel miRNAs) regulate the expression of 237 target genes (Supplementary Table S9). However, for the known miRNAs identified, a total of 3386 different targets conferring 840 unique descriptions targeting 1847 stress-associated genes (Supplementary Table S10). Detailed analysis of the target genes for known and novel miRNAs revealed that the majority of the targets were the genes for drought-responsive TFs including NB-ARC, WRKY, MYM, Zinc finger domains, Leucine-rich repeat domains, GRAS, NAC, MATH, WD 40, FAR1, RLCK, ARF, bZIP, MAPK, MADS, ARFs, and HSP, a few defense-related components (such as serine/threonine protein kinases, thioredoxin, peroxidase, and glutathione transferase), and transporters (including sodium, potassium, ABC transporters, glutathione-conjugate transporter, auxin, and sugar/inositol transporters, etc.). The same miRNA targeting multiple target genes and a gene targeted by several miRNAs were also noticed, indicating the interactions between miRNA and mRNAs under stress. Some of the important drought-responsive known miRNAs and their family as well as the target gene have been enlisted in Table 1.

### 2.5. Expression Analysis of miRNA Target Gene

**Figure 6:**
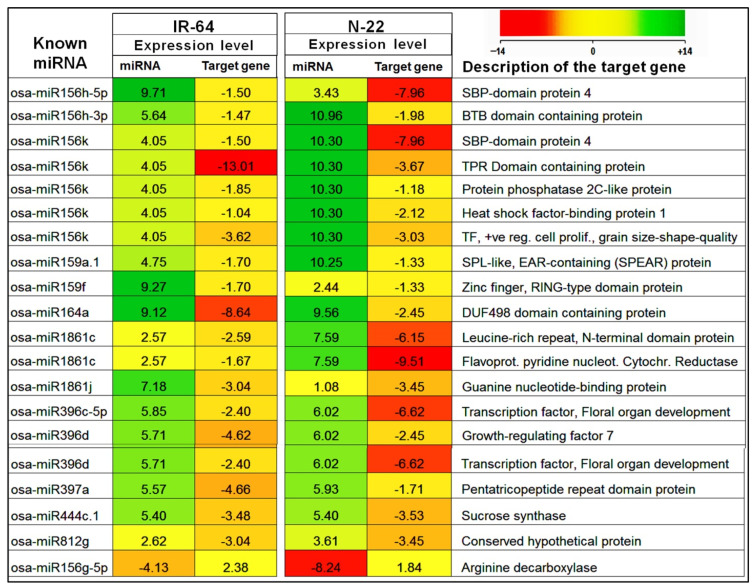
Heat map showing some of the differentially expressed known miRNAs and their effect on target gene expression in roots of contrasting rice cultivars imposed with reproductive stage (terminal) drought stress. The green color of the box (positive value) indicates up-regulated expression of miRNA/target gene. The red color of the box (negative value) indicates the down-regulated expression of the miRNA/target gene. The intensity of color corresponds to the magnitude of expression, the darker the shade indicates the stronger the expression and vice versa.

**Figure 7:**
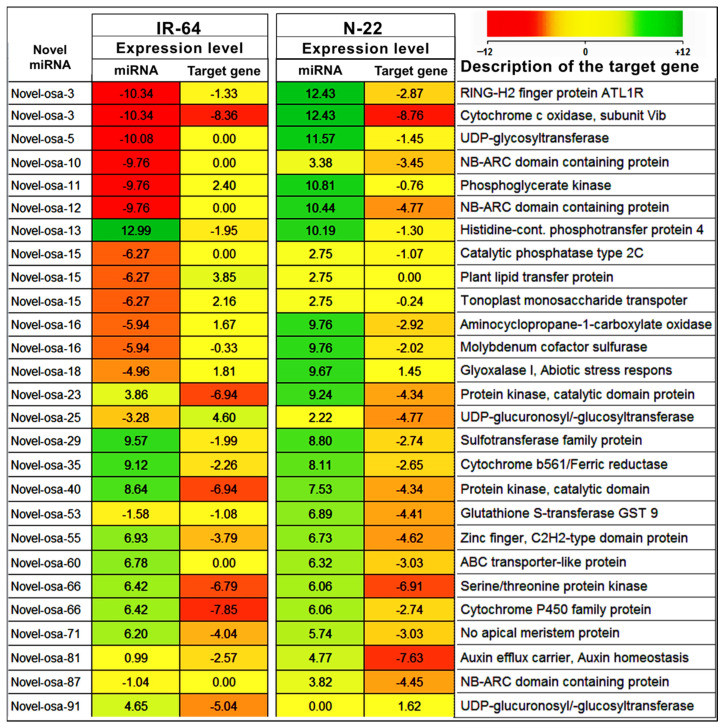
Heat map showing some of the differentially expressed novel miRNAs and their effect on target gene expression in roots of contrasting rice cultivars imposed with reproductive stage (terminal) drought stress. The green color of the box (positive value) indicates up-regulated expression of miRNA/target gene. The red color of the box (negative value) indicates the down-regulated expression of the miRNA/target gene. The intensity of color corresponds to the magnitude of expression, the darker the shade indicates the stronger the expression and vice versa.

In order to understand the impact of differentially expressed miRNAs on target gene expression, a comprehensive transcriptome analysis was conducted on the tissue samples. Using a >2-fold change and a P-adjusted cut-off value of 0.05, a total of 16,341 and 18,008 differentially expressed genes (DEGs) were identified in the N-22 and IR-64 rice cultivars, respectively. Significant up- or down-regulation of numerous genes was observed in a cultivar-specific manner, indicating the distinct activity of drought-responsive genes in response to reproductive stage drought stress (Supplementary Table S11). To investigate the influence of known and novel miRNAs on target gene expression, a comparison was made between the expression levels of the miRNAs and their target genes. The comparative analysis revealed that up-regulated expression of miRNAs corresponded to the down-regulation of their target genes. The effects of differentially expressed known and novel miRNAs on target gene expression are depicted in Figure 6 and Figure 7. These findings provide valuable insights into the regulatory mechanisms mediated by miRNAs in response to reproductive stage drought stress, shedding light on the intricate interplay between miRNAs and their target genes in rice.

Many of the known miRNAs showed considerably up-regulated expression in roots of N-22 cultivar under drought stress resulting in significantly more down-regulated expression of the target gene, compared to that observed in roots of IR-64 (Figure 6). Interestingly, many of the novel miRNAs showed up-regulated expression in roots of the N-22 cultivar under drought stress resulting in significantly down-regulated expression of the target gene. More interestingly, these novel miRNAs were down-regulated in roots of IR-64 under the stress with comparatively up-regulated expression of the target gene (Figure 7). Moreover, the correlation among the most significant pathways highly enriched in the contrasting rice cultivars in, response to the drought stress, was depicted by hierarchical clustering tree analysis for the target genes (Supplementary Figure S1).

### 2.6. Gene Ontology Network Analysis of miRNA Targeted Genes 

**Figure 8:**
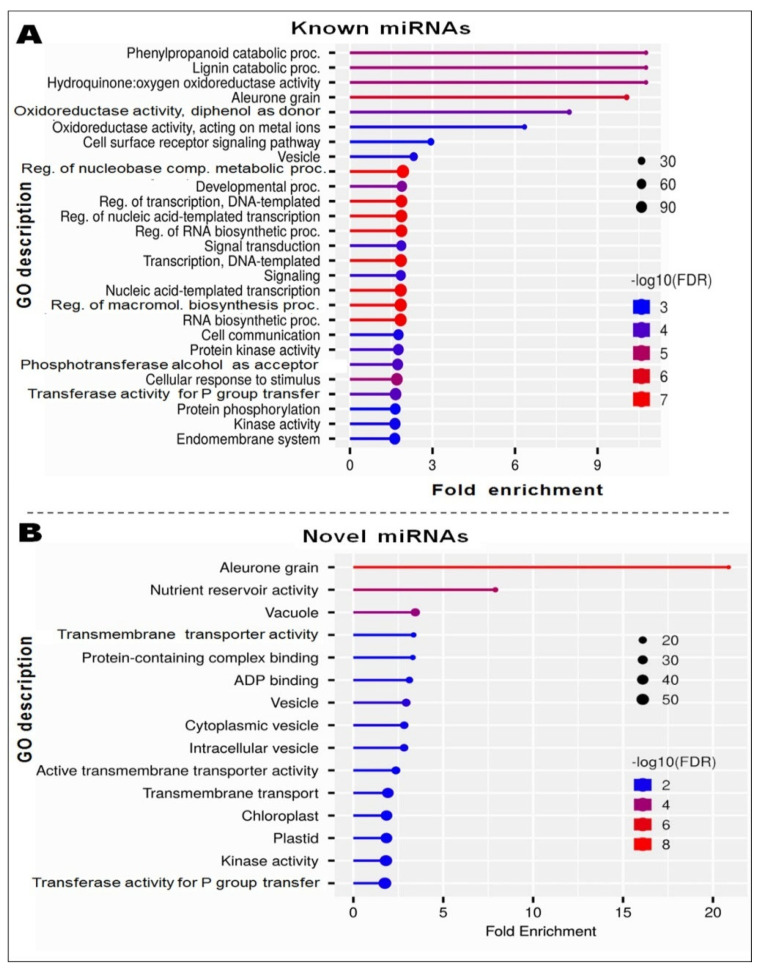
Gene ontology analysis of the targets of known and novel miRNAs in roots of the rice cultivars under control and reproductive stage (terminal) drought stress. (**A**) Highly enriched GO terms affected by the known miRNAs, (**B**) highly enriched GO terms affected by the novel miRNAs. The size of the lollypop indicates the respective gene count concerned with the specific GO term, while the color of the lollypop indicates the false discovery rate.

Based on the fold enrichment analysis (FDR ≤ 0.05), a total of 120 significantly enriched gene ontology (GO) terms for the targets of known miRNAs and 27 GO terms for the targets of novel miRNAs were identified (Supplementary Tables S12 and S13). The most significantly affected GO terms by the known miRNAs included those for phenylpropanoid catabolic process (GO:0046271), lignin catabolic process (GO:0046274), hydroquinone: oxidoreductase activity (GO:0052716), and Aleurone grain (GO:0033095) (Figure 8A). In the case of the novel miRNAs, the most significantly affected GO terms were those for aleurone grain (GO:0033095), nutrient reservoir activity (GO:0045735), vacuole (GO:0005773), and transmembrane transporter activity (GO:0015399) (Figure 8B).

**Figure 9:**
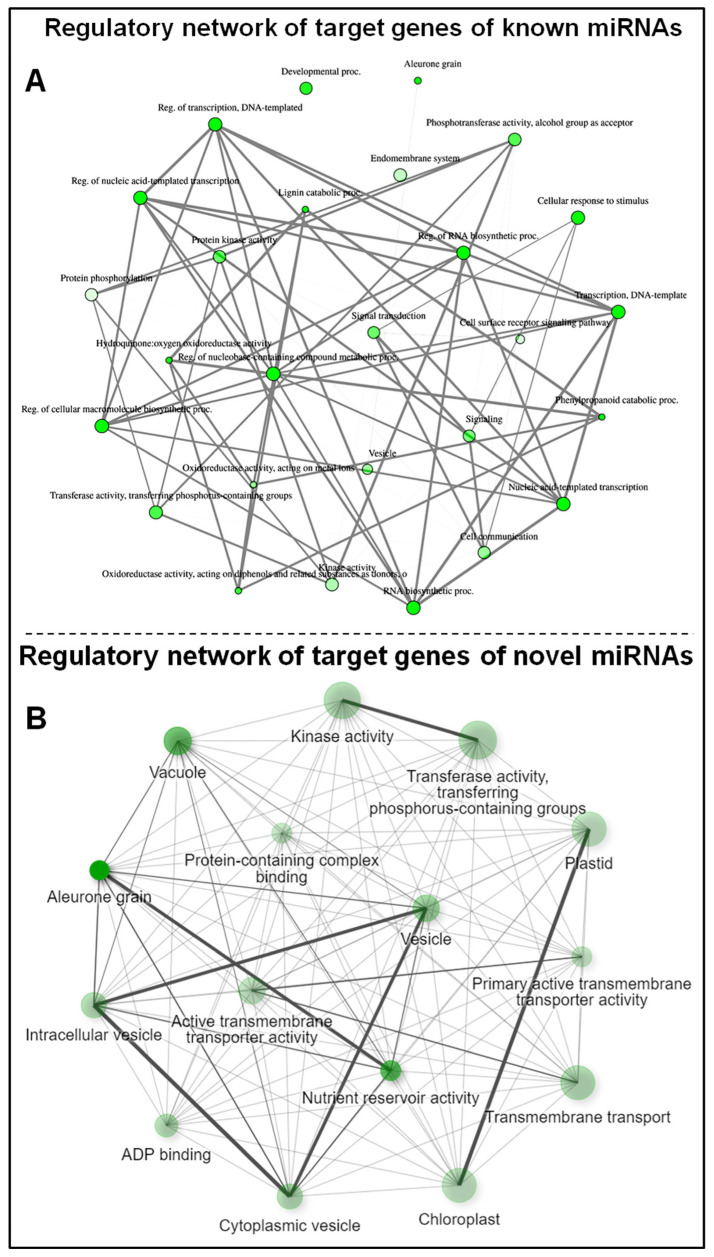
Gene regulatory network for the targets of (**A**) known and (**B**) novel miRNAs expressed in roots of the contrasting rice cultivars in response to reproductive stage drought stress. The darker nodes represent the highly enriched gene sets, the bigger the node depicts the larger gene sets, the thicker the edge indicates the more overlapped genes.

Gene network analysis for the significantly enriched GO terms depicted interactions between the significantly enriched GO terms affected by the known miRNAs (Figure 9A) and novel miRNAs (Figure 9B) in roots of contrasting rice cultivars imposed with terminal drought stress. Clustering analysis to depict the interactions among the pathways affected by known and novel miRNAs in the rice cultivars under terminal drought stress is shown in Figure 9.

### 2.7. Functional Validation of miRNAs by RT-qPCR Assay

**Figure 10:**
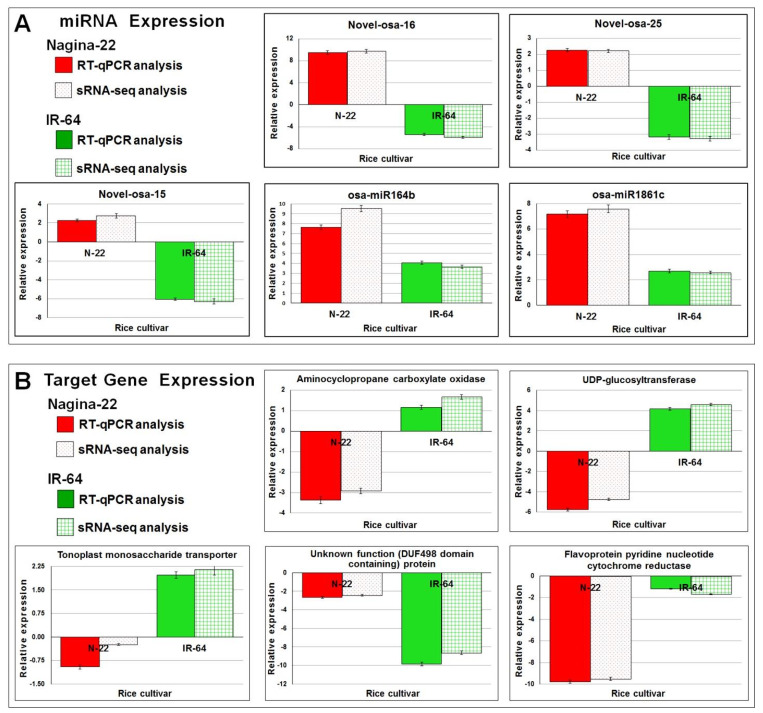
The validation of differential expression of miRNA and its target gene in roots of the contrasting rice cultivars. (**A**) The expression level of miRNAs observed on RT-qPCR and sRNA-seq analysis are presented, (**B**) RT-qPCR validation of the miRNA targeted gene expression in roots of the rice cultivars. Data represent the mean ± SD (*n* = 3).

To validate the accuracy of the sRNA-seq data and confirm the functional impact of miRNA on the target gene, reverse transcription-quantitative PCR (RT-qPCR) analysis was performed. This analysis focused on the expression levels of five differentially expressed novel and known miRNAs, which are responsive to drought stress, along with their target genes in root tissues. The RT-qPCR results demonstrated a similar expression pattern to that observed in the sRNA-seq and transcriptome analyses, providing validation for the reliability of the datasets (Figure 10). As anticipated, the up-regulated expression of miRNA under terminal drought stress led to a down-regulation of the corresponding target gene expression in the roots. Notably, a negative correlation between the expression levels of miRNA and the target gene was observed, particularly in the N-22 rice cultivar, under stress conditions. These findings provide functional evidence supporting the trustworthiness of the sRNA-seq and transcriptome datasets. The RT-qPCR analysis successfully validated the expression of miRNA and its influence on target gene degradation, further reinforcing the reliability of the obtained results.

## 3. Discussion

Among the various regulators of gene expression, differential expression of miRNA under abiotic stresses is reported to be a crucial player in plants. Several studies have deciphered the roles of miRNAs in regulating gene expression under different abiotic stresses [23,31,34,37,50,52,54,58,59,60,61,62,63]. Being a semi-aquatic/water-loving plant, the germination, growth, development, and productivity of rice are severely affected by drought stress [10]. Moreover, the reproductive stage (terminal) drought drastically reduces grain yield [8,9,10]. Therefore, to minimize the yield loss in this era of global climate change and frequent occurrence of drought stress (as well as other abiotic/biotic stresses), the need of the day is to develop rice cultivars with enhanced tolerance to multiple stresses (climate-smart rice) with better yield under the changing climatic conditions [10,64]. 

A number of drought-responsive miRNAs expressed in different tissues at different developmental stages and their target genes in rice have already been identified [4,5,25,29,31,34,42,54,65,66] suggesting that miRNAs play crucial roles in drought tolerance mechanisms. However, only a little is known about the miRNAs expressed under terminal drought stress in different tissues of rice, particularly in roots. Since the root is the preliminary tissue/organ responsible for water uptake/sensing drought stress, it requires several adaptive changes (alterations in RSA) to mitigate the deleterious effects of drought stress. Hence, one of the objectives of developing climate-smart rice should be to understand the molecular (genetic/epigenetic/miRN*omic*) basis of the genetic/physiological plasticity of rice, particularly in roots, under abiotic/biotic stresses [8,10,22]. Studies suggest that differential expression of drought-responsive miRNAs in root play vital roles in scavenging reactive oxygen species, defense signaling, auxin-abscisic acid crosstalk, formation of lateral roots, and leaf polarity [16,17,56,67]. While the roots play primary roles in mitigating the adverse effects of drought stress, the leaves help manage the survival of the plant under the stress [22].

In the present study, a comparative miRN*ome* analysis was carried out in roots of contrasting rice cultivars (IR-64 and N-22) grown under control and severe drought stress imposed at the reproductive stage of plant growth (Figure 1). The number of miRNAs (known as well as novel) differentially expressed under control and the stressed conditions in the rice cultivars (Figure 2) indicated their role in imparting stress tolerance. A large number of novel miRNAs, distributed throughout the genome, expressed under the terminal drought stress in rice (Figure 3) indicate that only a little information about the roles of miRNA is available so far. This view was further strengthened by the fact that >12% of the novel miRNAs were exclusively expressed in roots of N-22 under stress (Figure 4). Up-regulated expression of known (223) as well as novel (82) miRNAs play major roles in providing stress tolerance to N-22 (Figure 5). 

The target prediction analysis conducted for both known and novel miRNAs revealed that a significant proportion of these miRNAs targeted transcription factors responsible for regulating various biological processes during drought stress (Table 1). Furthermore, hierarchical clustering analysis of the target genes, targeted by known and novel miRNAs expressed in the roots of the contrasting rice cultivars under terminal drought stress, indicated distinct functional roles. The novel miRNAs were predominantly associated with controlling cellular/organellar functions, while the differentially expressed known miRNAs were found to be involved in regulating transcription/metabolic processes, stress signaling, phosphotransferase, oxidoreductase activities, and more, in response to drought stress in the contrasting rice cultivars (Supplementary Figure S1). The comprehensive analysis sheds light on the intricate network of miRNA-mediated gene regulation during drought stress in rice.

The miR166 family members have been reported to be involved in the regulation of class III homeodomain Leu-zipper (HD-Zip III) and OsHB4, which participate in the development of lateral roots, leaf polarity, controlling plant architecture, and leaf development [17,68,69,70]. Knocked out of miR166 in rice was reported to show responses similar to that of normal plants under drought [69]. Normally, the plants show rolling of leaves, decreased stomatal conductance, lower hydraulic conductivity, and reduced transpiration rate under drought resistance. Our observations on the differential expression of 20 different members of the miR166 family and two novel miRNAs (novel-osa-39 and 75), targeting the HD-Zip III gene involved in regulating root development, leaf architecture, and drought-tolerance in rice (Table 1, Supplementary Table S8) are in agreement with findings of Zhang et al. [69].

The interaction of miRNA with its target gene(s) is crucial because this helps determine the expression level of the drought-responsive gene(s). The miR156 was reported earlier to target squamosal promoter-binding-like (OsSPL) transcription activator, regulating shoot and plant architecture development as well as panicle branching in rice [71], which corroborates with our findings on differential expression of 19 different miRNA members of miR156 family and two (novel-osa-45 and 105) miRNAs, targeting SPL, under the stress. In alfalfa, overexpression of miR156 was reported to alter several physiological responses under drought stress and increase/decrease the expression of positive/negative regulator genes, respectively. Members of the miR156 family showed differential (up- and down-regulated) expression in roots of IR-64 and N-22 (Supplementary Tables S4 and S5), suggesting cultivar-specific regulatory effects of the miRNAs under the stress.

Studies suggest the role of auxin-ABA crosstalk under drought stress. miRNA167 targeting several auxin-response factors (ARFs) including ARF 6 and ARF 8. ARFs were reported to be down-regulated in rice [72] under drought stress. Down-regulated expression of miR167 under drought stress enhances the accumulation of phospholipase D, required for ABA signaling [27,73]. ARFs, NAC, and AGO1 are the TF families that regulate auxin-induced alterations in plant tissues [2,74]. Under drought stress, miR160 was reported to be down-regulated; hence, up-regulates expression of its target ARF10 provides drought stress tolerance [75]. Studies suggest that miR167 regulates ARF6 and ARF8 in rice [2,72,74,76,77]. We observed differential expression of 11 different miR167 family members along with varied expression of novel_miR-osa-4, -osa-10, -osa-12, -osa-60, -osa-63, and -osa-71 targeting NB-ARC domain-containing protein. Moreover, 5 different members of the miR160 family as well as novel_miR-osa-65, -osa-71, and -osa-81 were identified to regulate auxin response and transport (Table 1, Supplementary Table S9). Down-regulated expression of miR160 (a negative regulator of ARF 10 and 16) was proposed to play a crucial role in auxin-mediated increased length of roots under drought stress [67].

Increased expression of miR164 family members was reported to decrease the expression of several NAC TFs resulting in delayed senescence of leaves [2] and reduced tolerance to drought in rice [45]. Our findings on the differential expression of five members of the miR164 family and two novel miRNAs (novel_miR-osa-37 and novel_miR-osa-71) targeting NAC TFs in response to the drought stress corroborate the earlier reports and indicate the phytohormonal crosstalk in rice under drought stress (Table 1, Supplementary Table S9). Differential expression of seven members of the miR159 family, targeting GAMYB-like protein, has been reported to be involved in flower development and stem elongation at the reproductive stage [78]. Differential expression of miR393 family members, regulating the tiller number, early flowering, auxin-response, and horizontal root development (mainly by controlling *OsAUX1* and *OsTIR1*) during drought stress is in agreement with the earlier reports [35,79].

**Figure 11:**
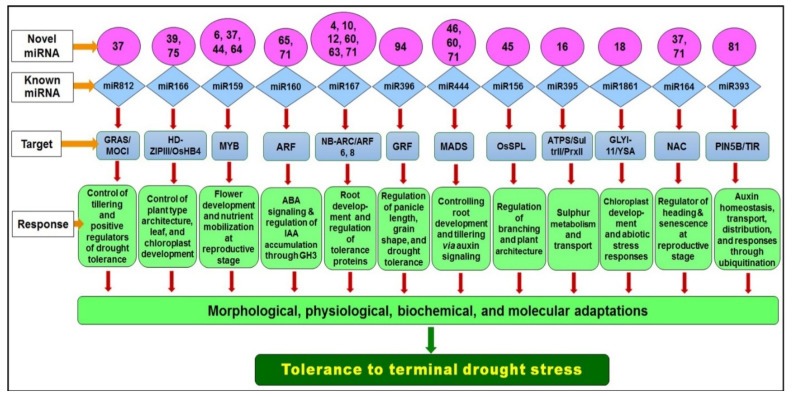
Pictorial presentation of the involvement of novel and known miRNA-mediated regulatory gene network under terminal drought stress in rice.

Validation of the functions of selected miRNAs on the degradation of mRNA from the target gene using transcriptome as well as RT-qPCR analysis (Figure 10) confirmed the important roles of known as well as newly identified miRNAs in tolerance to terminal drought stress. However, further validation of the functions of the miRNAs would be necessary through degradome analysis as well as genome editing/genetic manipulation approaches. Based on our findings, we propose a model for miRNA-mediated gene regulation and stress responses in rice on reproductive stage drought stress (Figure 11). Thus, our findings provide deeper insights into the involvement/role of miRNAs (and the targeted genes) under terminal drought stress in rice. The identified novel miRNAs expressed under terminal drought stress would not only enrich the miRBase dataset but some of them may serve as candidates for developing drought-tolerant climate-smart rice cultivars for sustainable food security.

## 4. Materials and Methods 

### 4.1. Plant Materials and Drought Stress Imposition

Mature and healthy seeds of two rice cultivars, Nagina-22 (drought-tolerant) and IR-64 (sensitive to reproductive stage drought) received from the Division of Genetics, ICAR-Indian Agricultural Research Institute, New Delhi, India, were used to grow rice in 12” pots inside a net-house at the Division of Biochemistry, ICAR-Indian Agricultural Research Institute in New Delhi, India. The plants were grown during the *Kharif* season under natural conditions. The experimental setup involved raising seedlings in a nursery and transplanting 25-day-old seedlings into pots filled with puddled soil. The pots were divided into two sets: one set was used for growing rice plants under control conditions, receiving irrigation on alternate days, while the other set was subjected to drought stress during the reproductive stage of plant growth, specifically at the initiation of flowering. The drought stress was induced by withholding irrigation for a period of 4–5 days just before panicle initiation. The intensity of the drought stress was determined by measuring the soil moisture content, which decreased to approximately 6% in comparison to the 24% moisture content in the control pots. Additionally, the relative water content of the leaves decreased to approximately 58%. Root tissues were collected from the control and drought-treated rice plants in nine replications for each condition, to analyze the impact of drought stress on roots. To collect root tissues, the plant was removed carefully from the pot along with the soil. The roots embedded in the soil were tapped gently with a wooden plank to loosen/pulverize the soil and removed to expose the roots. Roots were cleaned carefully to minimize the damage. The root tissues, thus exposed, were washed with ethanol four times to remove soil particles and any contaminants. After cleaning, the roots were placed between paper towels to blot dry the roots. Finally, the root tissues were wrapped in aluminum foil, dipped in liquid nitrogen, and stored at −80 °C in multiple replications for further downstream processing.

### 4.2. RNA Isolation, Small RNA Library Construction, and Sequencing

Total RNAs were isolated in six replications from the root tissues using TRIzol reagent following the protocol mentioned earlier [22]. The quality of the RNAs was checked by denaturing agarose gel electrophoresis and measuring the RIN value (>6.0). The smallRNA-seq libraries for IR-64 root control (IRC), IR-64 root drought treated (IRT), Nagina-22, root control (NRC), and Nagina-22 root drought treated (NRT) were prepared from the pooled total RNAs in two replications using Illumina TruSeq small RNA library preparation kit following manufacturer’s protocol. The libraries were sequenced on Illumina HiSeq 2500 platform using SE 50 chemistry in a single replication. The small RNA-seq data were submitted to NCBI (SUB12945434), which can also be made available on request.

### 4.3. Small RNA Sequencing Reads Analysis

Raw reads from a library were cleaned for contaminating sequences using Trim Galore software (https://www.bioinformatics.babraham.ac.uk/projects/trim_galore/, accessed on 2 Januray 2023) set at the following quality control (QC) threshold: (a) reads with Phred score 30; (b) reads contaminated with adaptor sequences; (c) reads with a length < 17 and >35 nt; (d) reads with an unknown base content (N) of >10%; (e) discarding the reads possessing a poly-A tail. The clean reads matching with the known t/rRNA sequence were discarded after identification by searching in the GenBank (www.ncbi.nlm.nih.gov/genbank/, accessed on 2 Januray 2023) as well as Rfam (https://rfam.xfam.org/, accessed on 2 Januray 2023) databases using SortMeRNA [80]. Further downstream analysis was performed using a 17–35 nt filter for small RNA reads. Filtered reads for the libraries were aligned with *the Oryza sativa* Japonica reference genome (https://plants.ensembl.org/Oryza_sativa/Info/Index, accessed on 2 Januray 2023) using Bowtie [81]. To identify known and novel miRNAs, the obtained sequences were aligned with the miRBase database (www.mirbase.org/, accessed on 2 Januray 2023), which contains a collection of annotated miRNAs. For the known miRNAs, sequences showing alignment with miRBase were considered. For the unannotated reads, which did not align with any miRBase sequences, miRDeep2 was employed to identify novel miRNAs [82]. The miRDeep2 software offers a comprehensive analysis of potential novel miRNAs from deep sequencing data. To align the reads with rice miRNAs from miRBase 22.1 [83], the mapperl.pl script was utilized. This script facilitates the alignment process, enabling the identification of potential miRNAs within the rice genome. Quantification of both known and novel miRNAs in the libraries was performed using the quantifier.pl script from miRdeep2. This script allows for accurate quantification of miRNA expression levels based on the obtained sequencing data. By combining these alignment and quantification approaches, the study aimed to identify and quantify both known and novel miRNAs in the analyzed libraries, enabling a comprehensive analysis of miRNA expression patterns.

### 4.4. Differential Expression of Known and Novel miRNAs 

The known and novel miRNA read counts were normalized using TPM [actual miRNA count ÷ total count of clean reads] × 10^6^, while miRNAs with TPM ≥ 1 in at least one of the libraries were selected for further analysis. Predicted novel miRNAs were filtered with the following cut-offs: a maximum of 4 mismatches were permitted between miRNA and star miRNA pairing; desired length of precursor miRNA should be less than 60; the A+U content of the precursor miRNA should be ≥30%; the minimal folding energy (MFE) of precursor miRNAs must be ≤−20 kcal/mol; the minimum free energy index (MFEI) should be ≥0.70; predicted miRNAs with significant randfold *p*-value; and no gaps or loops permitted in the miRNA and star miRNA duplex complex [84,85,86,87]. Differential expression analysis of the known and novel miRNAs was performed using EdgeR and differentially expressed miRNAs were filtered with a fold change of ≥2 among the libraries. The positive value for the expression of miRNAs/target genes represents up-regulation, while the negative value represents a down-regulated expression of miRNA/target gene under drought stress in the rice cultivars (Supplementary Tables S4–S8). A temporary name was assigned to the identified novel miRNAs, while the final name/number for the novel miRNA would be assigned by the miRBase Registry.

### 4.5. Target Gene Prediction and Functional Enrichment Analysis of Targets

**Figure 12:**
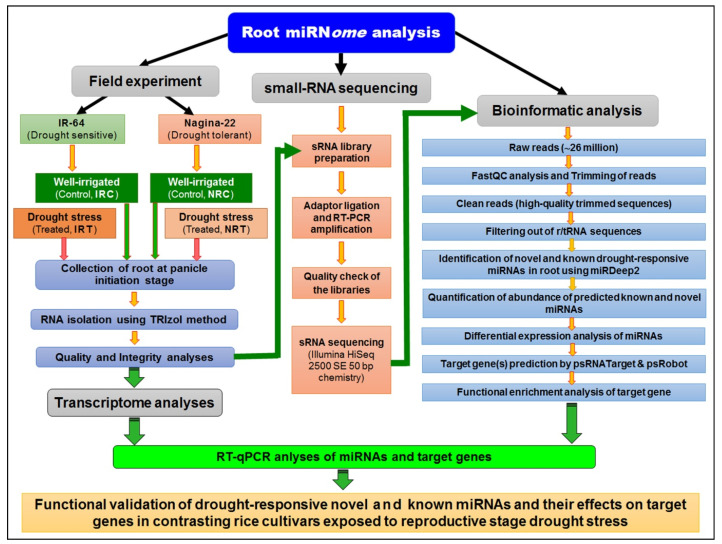
Overview of the methodology used for identification, characterization, and functional validation of the reproductive stage drought-responsive known and novel miRNAs in roots of rice.

The psRNA-Target program (http://plantgrn.noble.org/psRNATarget/, accessed on 10 Januray 2023) [88] was utilized with default parameters to predict target genes for both differentially expressed known and novel miRNAs. For target prediction, the following cut-off values were employed: a target accessibility (UPE) of 25.0, a flanking length of 17 around the target site for target accessibility, a maximum expectation of 3, a length of 20 for complementarity scoring, a penalty of 1 for mismatches, and a range of central mismatch of 9–11 nt leading to translational inhibition. GO enrichment analysis for the target genes of known and novel miRNAs was performed using ShinyGO v0.76.2 software (http://bioinformatics.sdstate.edu/go/, accessed on 10 Januray 2023). A false discovery rate (FDR) cut-off threshold of 0.05 was used for the GO analysis. The background list of genes and GO annotations were obtained from the Rice Genome Annotation Project (RGAP). Furthermore, gene regulatory network analysis and hierarchical clustering were performed to assess the correlation among the pathways targeted by known and novel miRNAs. The software used for generating these analyses is not specified. Figure 12 provides an overview of the miRNome analysis conducted under stress conditions, illustrating the key findings and insights obtained from the study. 

### 4.6. Whole-Genome Transcriptome Analysis for Functional Validation of miRNA

To assess the impact of differentially expressed miRNAs on target gene expression, total RNA was extracted in three replicates from the roots of rice plants cultivated under both control conditions and reproductive stage (terminal) drought stress. The TRIzol method, as described earlier, was employed for RNA isolation. To eliminate any DNA contamination, the RNA samples were treated with DNase I. The quality and integrity of the RNA were evaluated using a 1.2% denaturing agarose gel, while quantification was performed using the Qubit 4. Approximately 1.0 µg of high-quality RNA, meeting specific criteria such as OD 260/280 ratio of approximately 2.0, OD 260/230 ratio greater than or equal to 2.0, RNA Integrity Number (RIN) of at least 6.0, and 28S:18S ratio of at least 1.0, was utilized for the construction of RMA-seq libraries in three replicates. The library construction process involved mRNA enrichment, RNA fragmentation, first- and second-strand cDNA synthesis, purification, sequencing adaptor ligation, and PCR amplification, following the protocols provided in the TruSeq RNA Sample Preparation kit from Illumina. The resulting libraries were commercially sequenced using the Illumina HiSeq 2500 platform with PE 150 bp chemistry, conducted by Macrogen in South Korea. The raw RNA-seq data were submitted to the NCBI under the accession number SUB11354353, with selected BioSamples. These raw RNA-seq data, obtained from the sequencing process, were subsequently utilized for various bioinformatic analyses as part of our research.

The quality assessment of the raw reads was performed using FastQC, and subsequent read trimming was conducted using Trim Galore software. This trimming step involved the removal of adaptor sequences, poly-N stretches, and low-quality reads. The resulting clean reads were then aligned to the *Oryza sativa* Japonica reference genome IRGSP-1.0 using the STAR aligner (genome indexing followed by genome mapping while performing STAR) [89]. Feature counts were obtained using featureCounts [90] to assign the clean reads to genomic features. To ensure comparability among samples, the raw read counts were normalized using the reads per kilobase of exon per million mapped reads (RPKM) method. Differential expression analysis between different comparison groups was conducted using the DESeq2 package [91]. The resulting *p*-values were adjusted for multiple testing using the Benjamini and Hochberg method to estimate the false discovery rate (FDR). For the identification of differentially expressed genes (DEGs), genes with fold changes greater than 2 (|log2Ratio| > 1) and statistically significant abundance with FDR < 0.05 were considered [92] (Supplementary Table S11). To explore the effects and roles of miRNAs in drought stress tolerance, the expression levels of both miRNAs and their target genes were compared under stress conditions.

### 4.7. Validation of Differential Expression of miRNA by RT-qPCR

To validate the differential expression of miRNAs, we performed RT-qPCR analysis using total RNAs isolated from an independent set of samples obtained from the rice cultivars (N-22 and IR-64) grown under similar experimental conditions during the *Kharif* season in 2022. For the RT-qPCR analysis, RNA was poly-adenylated and reverse transcribed using the Mir-X miRNA First-Strand Synthesis kit (TaKaRa Bio, San Jose, CA, USA, Inc.) following the manufacturer’s instructions. The synthesis of cDNA involved a reaction mixture containing RNA (2 µg), mRQ Buffer (2×), and mRQ enzyme mix. The reaction mixture was incubated at 37 °C for one hour, followed by termination at 85 °C for 5 min. The resulting cDNA samples were diluted 10 times using RNase-free water. In the RT-qPCR validation, the entire sequence of the mature miRNA (21–23 nt) was used as the miRNA-specific 5′ primer, while the 3′ primer for qPCR was the mRQ 3′ primer supplied with the kit. The reaction mix for RT-qPCR consisted of cDNA, primers, mRQ 3′ Primer, TB Green Advantage qRT-PCR premix, and RNase-free water. The amplification was performed on a QIAquant 96 5plex machine (Qiagen, Germany) with a programmed thermal cycling profile. The delta-delta Ct method was used to determine the relative expression level of each miRNA, normalized to the expression of U6 snRNA, which served as the internal control primer supplied with the kit. Supplementary Table S14 provides the sequence details of the miRNA-specific forward primers, universal reverse primers, and U6 snRNA primers used for the RT-qPCR validation of miRNA expression.

### 4.8. RT-qPCR Validation of Target Gene Expression

To validate the miRNA-mediated regulation of target gene expression and ensure the reliability of the transcriptome data, we conducted RT-qPCR analysis using an independent set of samples obtained from the rice cultivars grown under similar experimental conditions during the *Kharif* season. For this analysis, gene-specific primers were designed using Primer3 Plus software. To initiate the analysis, 2 μg of total RNA isolated from the sample tissue was used to synthesize cDNA following the manufacturer’s instructions, specifically utilizing the Superscript III First-Strand Synthesis Kit from Invitrogen. The resulting cDNA was then diluted five times and utilized as a template for the qPCR validation of the target genes. The analysis was performed using the QIAquant 96 5plex system from Qiagen, employing 10 µL of KAPA SYBR fast mix. The thermal cycler was programmed with an initial denaturation step at 95 °C for 20 s, followed by 40 cycles consisting of denaturation at 95 °C for 3 s, annealing at 60 °C for 20 s, and extension at 72 °C for 40 s. Amplification data was collected at the end of each extension step. Melt curve analysis was conducted to verify the specificity of PCR amplification. The relative expression of the target genes was determined using the 2^−ΔΔCT^ method, with the housekeeping/internal control genes Actin (LOC_Os03g50885) and β-tubulin (LOC_Os01g59150) being utilized. The primer sequences are specific to the target genes for the validation of their expression can be found in Supplementary Table S14.

## 5. Conclusions

Yield loss in crops due to the recurrent occurrence of drought stress has dramatically increased with changing global climate. To ensure sustainable food security, it is crucial to enhance the ability of crop plants to withstand both abiotic and biotic stresses. Our study has identified a substantial number of differentially expressed miRNAs, including known (270) and novel (91) miRNAs, in the roots of rice under the reproductive stage and terminal drought stress conditions. Notably, a significant proportion of these miRNAs were found to target drought-responsive transcription factors (TFs) and transporters. These identified miRNAs represent promising candidate genes for improving drought stress tolerance in rice. Of particular interest, our target prediction analysis revealed that certain miRNAs have the potential to target multiple genes. This characteristic makes them particularly valuable for genetic manipulation and engineering strategies aimed at enhancing stress tolerance in crop plants. Many of the identified miRNAs were predicted to target the genes involved in root development, phytohormone crosstalk, antioxidant defense, plant architecture, flower, and grain development. Validation of the expression pattern of a few selected miRNAs and their target genes, using RT-qPCR analysis, in an independent set of tissue samples ensured the functionality of the miRNAs. We believe that our findings would not only enrich the miRBase database but also help design climate-smart, sustainable rice for global food security in this era of climate change. Lastly, by harnessing the regulatory potential of these differentially expressed miRNAs, especially those targeting TFs and transporters, we can advance efforts to develop drought-tolerant rice varieties. These findings offer valuable insights into the genetic basis of drought stress response and pave the way for future research on improving stress tolerance in crop plants through miRNA-mediated genetic manipulation.

## Figures and Tables

**Table 1:** List of the drought-responsive known miRNAs and their target genes identified in root tissue in rice.

miRNA Family	Known miRNAs (Total Count)	Description of Target Gene
miR812	osa-miR812a, b, c, d, e, f, g, h, i, j, k, l, m, n-5p, n-3p, o-5p, o-3p, p, s, t, u, and v (22)	GRAS family nuclear protein, Control of tillering (MOCI), Similar to serine/threonine protein kinase (OsCIPK10), Leucine-rich repeat, plant-specific protein, Similar to Na+/H+ antiporter, probable CP0838, Similar to 1-aminocyclopropane-1-carboxylate oxidase (Fragment) (ACO3), Similar to PDR-like ABC transporter (PDR4 ABC transporter).
miR166	osa-miR166a-5p, a-3p, b-5p, b-3p, c-5p, c-3p, d-5p, d-3p, e-5p, f, g-3p, h-5p, h-3p, i-3p, j-5p, j-3p, k-3p, l-5p, l-3p, and m (20)	Class III homeodomain Leu zipper (HD-Zip III) family member, Control of plant type architecture and leaf development (OsHB4), Leucine-rich repeat, N-terminal domain-containing protein, Similar to Rolled leaf1 (OsHox1), Serine/threonine protein kinase domain-containing protein (OsWAK105), Chloroplast-targeted Deg protease protein, Chloroplast development and maintenance of PSII function under high temperature (OsDeg10).
miR156	osa-miR156a, b-5p, b-3p, c-5p, c-3p, d, e, f-5p, f-3p, g-5p, g-3p, h-5p, h-3p, i, j-5p, j-3p, k, l-5p, and l-3p (19)	Positive regulator of cell proliferation, Control of grain size, shape, and quality (GW8), Squamosa promoter-binding-like transcription activator, Regulation of branching in panicles and vegetative shoots, Semi-dominant regulator of plant architecture (OsSPL).
miR1861	osa-miR1861b, c, e, f, g, h, I, j, k, l, and m (11)	Glyoxalase I, Abiotic stress response (GLYI-11), Glycoside hydrolase, carbohydrate-binding domain-containing protein, pentatricopeptide repeat protein, Chloroplast development (YSA), Similar to guanine nucleotide-binding protein α-1 subunit, Similar to GTP-binding protein-like; root hair defective 3 protein-like, Leucine-rich repeat, N-terminal domain-containing protein.
miR167	osa-miR167 a-5p, b, c-5p, d-5p, e-5p, f, g, h-5p, h-3p, i-5p, and j (11)	NB-ARC domain-containing protein, Similar to glutamate-gated kainate-type ion channel receptor subunit GluR5, pentatricopeptide repeat domain-containing protein, Short-chain dehydrogenase/reductase SDR family protein.
miR396	osa-miR396 a-5p, a-3p, b-5p, b-3p, c-5p, d, e-5p, f-5p, g, and h (10)	Growth regulating factor members (OsGRF), WD40/YVTN repeat-like domain-containing protein, Transcription activator, Gibberellin (GA)-induced stem elongation, Growth-regulating factor, Regulation of grain shape and panicle length, Negative regulation of seed shattering, Auxin efflux carrier protein, Auxin transport, Drought tolerance
miR444	osa-miR444 a-3p.2, b.2, b.1, c.2, c.1, d.3, d.2, e, and f (9)	MADS-box transcription factor, Cold tolerance, Control of tillering (OsMADS57), MADS-box transcription factor, Homologue of the AGL17-clade MADS-box genes, Regulation of root system development via auxin signaling (OsMADS25), Zinc finger, RING/FYVE/PHD-type domain-containing protein, pentatricopeptide repeat domain-containing protein.
miR159	osa-miR159a.2, a.1, b, c, d, e, and f (7)	GAMYB-like protein, Flower development and stem elongation at the reproductive stage (OsGAMYBL1), Transcriptional activator of gibberellin-dependent α-amylase expression, Regulation of nutrient mobilization in germination (GAMYB), Similar to Coatomer protein complex, β prime; β′-COP protein (OsWD40-125).
miR395	osa-miR395b, d, e, g, s, and y (6)	Similar to ATP sulfurylase (OsATPS), Similar to low-affinity sulfate transporter 3 (OsSultr2), Similar to Thioredoxin peroxidase (OsPrxII), Cytochrome b5 domain-containing protein (OsMSBP2), Sulphate permease.
miR160	osa-miR160a-5p, b-5p, c-5p, d-5p, and e-5p (5)	Auxin response factors (OsARF10, 13), Transcriptional factor B3 family protein (OsARF22).
miR164	osa-miR164a, b, d, e, and f (5)	NAC transcription factor is a positive regulator of heading and senescence during the reproductive phase (OsY37). Sugar transporter protein, Similar to Pollen-specific kinase partner protein.
miR393	osa-miR393a and b-5p (2)	Auxin receptor, Flag leaf inclination, Primary root growth, Crown root initiation, Seed development, Tillering (OsAFB2), F-Box auxin receptor protein, Nuclear protein, Flag leaf inclination, Primary root growth, Crown root initiation, Seed development, Tillering (TIR1), Protein kinase, catalytic domain-containing protein (OsWAK7/8).
miR397	osa-miR397a and b (2)	Heat shock protein (HSP40), Putative tetratricopeptide repeat (TPR)-containing protein, Growth and development, salt tolerance, abiotic stress tolerance (OsHsp40), Laccase (OsLAC2, 5, 7, 9, and 29).
miR398	osa-miR398a and b (2)	Similar to Superoxide dismutase [Cu-Zn] (Cu/Zn-SOD), Selenium binding protein.
miR408	osa-miR408-5p and 3p (2)	Similar to Auxin-responsive protein (Aux/IAA) (Fragment) (OsIAA30), Cupredoxin domain-containing protein (OsUCL30).

## Data Availability

Sequencing data for sRNA libraries (submitted to NCBI, SUB12945434) will be made available on request. The RNA-Seq (transcriptome) data (submitted to NCBI) are available under the BioProject Submission ID SUB11354353.

## Supplementary Materials

The following supporting information can be downloaded at: https://www.mdpi.com/article/10.3390/genes14071390/s1, Figure S1: Hierarchical clustering tree for the target genes of (A) known and (B) novel miRNAs identified in roots of contrasting rice cultivars in response to terminal drought stress; Table S1: Mapping statistics of sRNA-seq data for roots from N-22 (drought-tolerant) and IR-64 (drought-sensitive) rice cultivars grown under control and terminal drought stress; Table S2: Transcripts per million (TPM) count for known microRNAs identified in the contrasting rice cultivars grown under control and terminal drought stress; Table S3: Novel microRNAs identified to be expressed in the contrasting rice cultivars grown under control and drought stress conditions; Table S4: Up-regulated expression of known miRNAs in roots of N-22 under terminal drought stress; Table S5: Down-regulated expression of known miRNAs in roots of IR-64 under terminal drought stress; Table S6: Up-regulated expression of known miRNAs in roots of IR-64 under terminal drought stress; Table S7: Down-regulated expression of known miRNAs in N-22 under terminal drought stress; Table S8: Differential expression of drought-responsive novel miRNAs in contrasting rice cultivars under terminal drought stress; Table S9: Target genes of the novel miRNAs in rice cultivars; Table S10: Target genes of the known miRNAs expressed in roots of rice cultivars; Table S11: Master data sheet for differentially expressed genes in contrasting rice cultivars grown under reproductive stage drought stress; Table S12: Gene Ontology enrichment analysis of known miRNAs targets in rice cultivars under terminal drought stress; Table S13: Gene Ontology enrichment analysis of novel miRNAs targets in rice cultivars under terminal drought stress; Table S14: List of primers used for RT-qPCR validation of selected novel miRNAs and their target genes differentially expressed in roots of the contrasting rice cultivars.
