# Supplementary material for: Comparative Genome-Wide Analysis of MicroRNAs and Their Target Genes in Roots of Contrasting Indica Rice Cultivars under Reproductive-Stage Drought
Source: Genes (Basel). 2023 Jul 1;14(7):1390. doi: 10.3390/genes14071390 (PMC10379292; doi:10.3390/genes14071390)

Supplementary Figure S1: Hierarchical clustering tree for the target genes of (**A**) known and (**B**) novel miRNAs identified in roots of contrasting rice cultivars in response to terminal drought stress. The correlation among the most significant pathways highly enriched in contrasting rice cultivars in response to the drought stress is depicted by clustering tree. Size of the dots signifies the *P*-value, bigger the dot more is the significance.

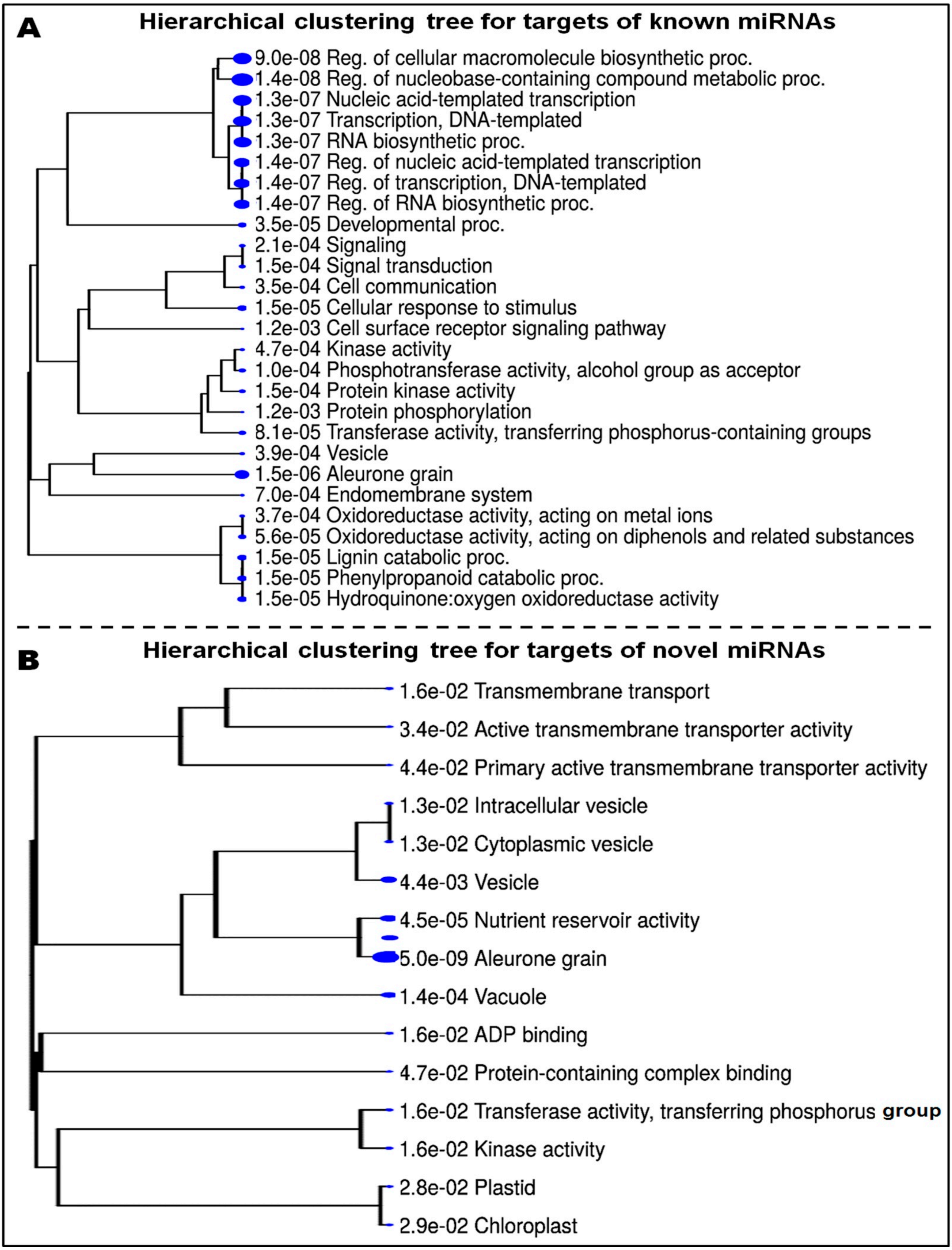

Supplement: Supplementary file 1 [file genes-14-01390-s001.zip › Supplementary Figure S1.pdf]
