# Supplementary material for: Comparative Genome-Wide Analysis of MicroRNAs and Their Target Genes in Roots of Contrasting Indica Rice Cultivars under Reproductive-Stage Drought
Source: Genes (Basel). 2023 Jul 1;14(7):1390. doi: 10.3390/genes14071390 (PMC10379292; doi:10.3390/genes14071390)
Supplement: Supplementary file 1 [file genes-14-01390-s001.zip › Supplementary Table S1.pdf]

Supplementary Table S1: Mapping statistics of sRNA-seq data for roots from N-22 (drought-tolerant) and IR-64 (drought-sensitive) rice cultivars grown under control and terminal drought stress. Number of miRNAs was predicted based on TPM count >1 in at least one of the samples.

Sample name	Sample description	Raw reads	Reads after trimming	Filtered reads (using SortMeRNA)
IRC	IR-64, Root, Control	33,435,457	8,660,128	8,569,730
IRT	IR-64, Root, Treated	23,648,557	4,034,879	4,586,589
NRC	N-22, Root, Control	27,551,686	8,928,014	8,856,851
NRT	N-22, Root, Treated	20,726,776	4,780,328	3,723,786
