# Supplementary material for: Comparative Genome-Wide Analysis of MicroRNAs and Their Target Genes in Roots of Contrasting Indica Rice Cultivars under Reproductive-Stage Drought
Source: Genes (Basel). 2023 Jul 1;14(7):1390. doi: 10.3390/genes14071390 (PMC10379292; doi:10.3390/genes14071390)
Supplement: Supplementary file 1 [file genes-14-01390-s001.zip › Supplementary Table S14.pdf]

Supplementary Table S14: List of primers used for RT-qPCR validation of selected novel miRNAs and their target genes differentially expressed in roots of the contrasting rice (IR-64, reproductive stage drought sensitive; N-22, drought tolerant) cultivars.

**Novel miRNA**		
**Name of miRNA**	**Targeted gene**	**Forward Primer** (5′→ 3′)
Novel-osa-16	1-aminocyclopropane-1-carboxylate oxidase	AUUUGUUGUAUUAGGGAAUGUCUCG
Novel-osa-15	Cytokinin-O-glucosyltransferase 1	UAGGUUUGUUUAUUUUGGGACG
Novel-osa-25	Transporter family protein	UGGAAAAUGCUAGAAUGACU
osa-miR156f-5p	SBP-domain protein 4	UGACAGAAGAGAGUGAGCAC
osa-miR164b	Protein of unknown function, DUF498 domain containing protein	UGGAGAAGCAGGGCACGUGCA
osa-miR1861c	Flavoprotein pyridine nucleotide cytochrome reductase domain containing protein	CGAUCUUGUAGCAAGAACUGAG
Universal Reverse Primer	3′ (reverse) primer supplied with the kit (Mir-X miRNA First-Strand Synthesis kit, Takara Bio USA, Inc.).	GTGCAGGGTCCGAGGT
U6F	The primer used as internal control (forward primer) supplied with the kit (Mir-X miRNA First-Strand Synthesis kit, Takara Bio USA, Inc.)	CAACGGATATCTCGGCTCT
U6R	The primer used as internal control (reverse primer) supplied with the kit (Mir-X miRNA First-Strand Synthesis kit, Takara Bio USA, Inc.)	CAACGGATATCTCGGCTCT

**Target gene** (of the identified novel miRNA)			
**Gene Name**	**Gene ID**	**Forward Primer** (5′→ 3′)	**Reverse Primer** (5′→ 3′)
1-aminocyclopropane-1-carboxylate oxidase protein, putative, expressed	LOC_Os02g53180	TGAAGCTCTCCATCCAGTCA	GACTCCGACTCCGACGAC
cytokinin-O-glucosyltransferase 1, putative, expressed	LOC_Os04g20400	TACCAGCGAGCAAGATGATG	TTGTGTGGTGCAGAAGAAGG
transporter family protein, putative, expressed	LOC_Os10g39440	AGCCGTCTGGCTACGACTAC	GCGTGGAGTCCCTGATCTC
Similar to SBP-domain protein 4	LOC_Os09g32944	AAGCCACAGGCAGATAGCAT	AAGCCACAGGCAGATAGCAT
Protein of unknown function DUF498 domain containing protein	LOC_Os06g20110	GCTCTGGTCTTGGAGGAGTG	GCTCTGGTCTTGGAGGAGTG
Flavoprotein pyridine nucleotide cytochrome reductase domain containing protein	LOC_Os05g33690	TCGACACGTGCTACAACCTC	TCGACACGTGCTACAACCTC
Actin gene	LOC_Os03g50885	TTGCTGACAGGATGAGCAAG	TGGAATGTGCTGAGAGATGC
β-tubulin gene	LOC_Os01g59150	GCTGACCACACCTAGCTTTGG	AGGGAACCTTAGGCAGCATGT
